# Changes in inflammatory responses and autophagy during apheresis platelet preservation and their correlation with platelet transfusion refractoriness in patients with acute lymphoblastic leukemia

**DOI:** 10.17305/bb.2023.9216

**Published:** 2023-12-01

**Authors:** Ying Li, Zhiqun Song, Xiaohong Sun, Juanjuan Tang, Xiaoyu Zhou

**Affiliations:** 1 Blood Transfusion Centre, The First Affiliated Hospital of Nanjing Medical University, Nanjing, China; 2 School of Medicine and Holistic Integrative Medicine, Nanjing University of Chinese Medicine, Nanjing, China

**Keywords:** Apheresis platelets (APs), platelet transfusion, platelet transfusion refractoriness (PTR), acute lymphoblastic leukemia (ALL), inflammatory response, autophagy, platelet preservation, peripheral blood mononuclear cells (PBMCs)

## Abstract

Acute lymphoblastic leukemia (ALL) is a common hematopoietic malignancy, and platelet transfusion plays a crucial role in its treatment. This study aimed to investigate the changes in inflammatory response and autophagy during the preservation of apheresis platelets (APs) and their correlation with platelet transfusion refractoriness (PTR) in ALL. ALL patients were included, and APs were categorized based on the preservation period (day 0, day 1, days 2–3, and days 4–5). The activation factors procaspase-activating compound 1 (PAC-1) and P-selectin (CD62P), AP aggregation function, inflammation levels (interleukin 1 beta [IL-1β], interleukin 6 [IL-6], tumor necrosis factor-alpha [TNF-α] and NOD-like receptor thermal protein domain associated protein 3 [*NLRP3*]), and autophagy-related genes (*p62*) during AP preservation, were assessed. Following co-culturing APs with peripheral blood mononuclear cells (PBMCs), specific activation markers were studied to observe APs influence on immune cells activation. The effectiveness of platelet transfusion was assessed, and risk factors for PTR were analyzed. As the storage duration of AP increased, the activation factors, coagulation factor activity, inflammation levels, and the activation of immune cells in AP increased, while fibrinogen levels and AP aggregation function decreased. The expression levels of autophagy-related genes (the autophagy marker light chain 3B gene [*LC3B*] and Beclin 1 gene [*BECN1*]) decreased with prolongation preservation. The effective rate of AP transfusion in ALL patients was 68.21%. AP preservation time, IL-6, *p62*, and *BECN1* were identified as independent risk factors affecting PTR in ALL patients. In conclusion, during AP preservation, inflammation, autophagy, and activation of immune cells were observed to increase. AP preservation time, IL-6, *p62*, and *BECN1* were independent risk factors for PTR.

## Introduction

Acute lymphoblastic leukemia (ALL) is the most common malignant tumor in children, arising from lymphoid hematopoietic stem cells in the bone marrow [1]. This clonal hematopoietic tumor is characterized by the proliferation and accumulation of hematopoietic progenitor cells, leading to impaired blood production and infiltration into major organs [2]. The effects of ALL extend beyond the bone marrow, with leukemia cells invading lymph nodes, liver, spleen, and other tissues, causing thrombocytopenia and bleeding complications [3]. Additionally, the administration of chemotherapy further contributes to bone marrow suppression and reduces platelet counts [4]. Therefore, platelet transfusion plays a vital role in the treatment of ALL, especially in managing thrombocytopenia, a common clinical symptom of hematologic malignancies [5].

Apheresis platelet (AP) transfusion, as a potentially life-saving treatment, is commonly employed to address bleeding complications associated with thrombocytopenia [6]. APs, obtained from a single donor, possess high platelet concentration, purity, and allow precise control of the infusion dose [7, 8]. Furthermore, AP transfusion reduces the risk of adverse reactions compared to whole blood transfusions [9] and facilitates accurate measurement of platelet counts, thus optimizing clinical utilization and conserving blood resources [10–12]. Moreover, AP donation results in minimal loss of red blood cells and white blood cells in donors, with rapid recovery and limited impact on the hematopoietic and coagulation systems [13, 14]. Nonetheless, the use of APs also presents certain disadvantages. During storage, platelet morphology and function may undergo changes, affecting their quality and clinical efficacy [15]. Additionally, platelet-related complications, including adverse reactions (such as fever and inflammation), platelet transfusion refractoriness (PTR), and life-threatening complications, can occur following transfusion [16, 17].

PTR refers to a significant decrease in platelet count following transfusion, which is a common complication observed in patients receiving multiple transfusions, particularly those with hematologic malignancies [18, 19]. As platelet transfusion becomes increasingly prevalent in clinical practice, the incidence of PTR has shown a notable upward trend, yet its underlying pathogenesis remains largely unclear [20]. Existing studies have implicated immunological factors (such as human leukocyte antigen alloimmunity and lymphocytotoxic antibodies) as well as non-immunological factors (including infection, high fever, sepsis, and graft-versus-host disease) in the development of PTR [21, 22]. However, limited research has explored the relationship between inflammatory factors and autophagy changes in APs and PTR in ALL patients during the preservation period. Therefore, this study aimed to investigate alterations in inflammatory response and autophagy levels during the preservation period of APs and their potential association with PTR in ALL patients. The findings of this study may contribute to a better understanding of PTR and provide a theoretical basis for its reduction.

## Materials and methods

### Study subjects

A total of 280 patients with ALL, who were diagnosed and treated at the First Affiliated Hospital of Nanjing Medical University between January 2021 and December 2021 and received at least one AP infusion during their treatment, were included in the study. Among the participants, there were 160 males and 120 females. In cases where a patient received multiple platelet transfusions, only data from the last transfusion were considered. The age of the patients ranged from 20 to 55 years, with a mean age of 39.33 ± 8.85 years. The inclusion criteria were as follows: (1) patients newly diagnosed and categorized according to the 2016 World Health Organization classification of myeloid neoplasms and acute leukemia [23], with diagnosis based on cytomorphology, immunophenotype, cytogenetics, and gene diagnosis; (2) clinical examination confirming the need for platelet infusion therapy in ALL patients, regardless of bleeding, with a platelet count < 20 × 10^9^/L; (3) age > 18 years; (4) availability of complete clinical records. The exclusion criteria were as follows: (1) presence of other blood system diseases; (2) prior platelet transfusion before diagnosis; (3) presence of infectious diseases; (4) presence of malignant tumor diseases; (5) presence of comorbidities that could affect the study, such as mental illness or heart disease; (6) missing clinical data, transfer to another facility, lost follow-up or voluntary withdrawal from the study.

### Origin and grouping of apheresis platelets

The APs used in this study were obtained from the blood center of the First Affiliated Hospital of Nanjing Medical University. A total of 44 blood donors (22 males and 22 females) participated, with ages ranging from 18 to 44 years (mean age 31.66 ± 6.95 years old). The selection of blood donors and the standards for their physical examination and blood tests followed the “Blood Donor Health Examination Standards.” The AP products were filtered to remove 99.99% of leukocytes and irradiated with ∼ Cesium-137 γ ray. Each unit (bag) of AP product contained a therapeutic amount for clinical platelet infusion, with a volume of 200–250 mL and platelet content ≥ 2.5 × 10^11^. The AP samples from different blood donors were stored for 5 days at 22 ^∘^C ± 2 ^∘^C under gently oscillating conditions. Platelet samples from different donors were randomly collected. The APs were grouped based on the storage period into the day 0 (d0) group, day 1 (d1) group, days 2–3 (d2-3) group, and days 4–5 (d4-5) group. The d0 group had a storage time of 0–4 h, with a total of 66 cases. The d1 group had a storage time of 4 h–1 d, with a total of 78 cases. The d2-3 group had a preservation time of 1 d–3 d, with 74 cases in total. The d4-5 group had a preservation time of 3 d–5 d, with 62 cases in total.

### Detection of procaspase-activating compound 1 (PAC-1) and P-selectin (CD62P)

To assess platelet activation, 1 µL of platelets, 100 µL of phosphate-buffered saline (PBS), 10 µL of fluorescence antibody PAC-1-fluorescein isothiocyanate (FITC) (No. 8088576, clone number: PAC-1, BD Company, NJ, USA), and 2 µL of CD62P-R-phycoerythrin (PE)-Cy5 (No. 8088576, clone number: AK-4, BD Company) were mixed in a flow tube and incubated at room temperature for 15 min in the dark. Subsequently, 1% paraformaldehyde (400 µL) was added to the tube and further incubated in the dark at room temperature for 10 min. The samples were then centrifuged at 400 *g* for 5 min, and the supernatant was discarded. The samples were re-suspended in ~500 µL of PBS and analyzed using the FACS Canto II flow cytometer (BD Biosciences, Franklin Lakes, NJ, USA). An identical IgG isotype control antibody, labeled with the same fluorescence, was used as a reference with fresh inactivated platelets under the same conditions to set the dot plot quadrant. The optical signals collected by the forward scattered light detector, side scattered light detector, and fluorescence detector were converted into voltage pulses and then into digital signals that could be stored and processed by a computer through an analog-to-digital converter. The data obtained from the flow cytometer were stored in flow cytometry standard (FCS) format, which included three files: sample acquisition file, data setup file, and data analysis result. The single-cell analysis of four parameters (forward scatter characteristics [FSC], side scatter characteristics [SSC], FITC, and PE) generated 8-bit data. The FCS data file was approximately 80 kB when 10,000 platelets were collected. Once the data was collected, cell subpopulations could be displayed in different formats. Histograms were used to display single parameters, such as FITC or PE, with the horizontal axis indicating the fluorescent channel and the vertical axis indicating the number of cells collected within that channel. Based on the single-parameter histogram, a statistical table of the data was created to output the results. The level of PAC-1 and CD62P was determined by calculating the percentage of PAC-1 and CD62P-positive platelets in the platelet population.

### Determination of thrombelastogram (TEG) parameters

The TEG5000 analyzer and platelet localization kits (Haemonetics, MA, USA) were used to measure the samples according to the manufacturer’s instructions. Briefly, 1000 µL of platelet samples were added to kaolin activator tubes and mixed thoroughly. Then, 340 µL platelet samples were collected and added to the test cup. Subsequently, 20 µL of 0.2 mol/L CaCl_2_ was added, the cup holder was raised, and the test rod was moved to the test position for detection. The test was conducted at 37 ^∘^C for 1 h.

### Enzyme-linked immunosorbent assay (ELISA)

The levels of inflammatory factors, including interleukin 1 beta (IL-1β) (cat. DLB50), interleukin 6 (IL-6) (cat. D6050), and tumor necrosis factor-alpha (TNF-α) (cat. DTA00D), were measured using ELISA kits (Quantikine, R&D Systems, MN, USA) following the kit instructions. Briefly, 100 µL of antibody was added to the reaction plate, mixed, and incubated overnight at room temperature. The reaction plate was washed three times with washing solution before adding the standard or platelets. After the incubation, the petri dish was rinsed three times. Then, 100 mL of conjugate was added and incubated at room temperature for 60 min, followed by three washes. Subsequently, 100 mL of substrate was added and incubated in darkness at room temperature for 15 min. Finally, 50 mL of stop solution was added to terminate the reaction, and the color was measured using an automated microplate spectrophotometer (Microplate Reader/Model 3550, Bio-Rad, CA, USA). The results were calculated using standard curves created in each test.

### Reverse transcription quantitative polymerase chain reaction (RT-qPCR)

Total RNA was extracted and isolated from platelet samples using the TRIzol reagent (Thermo Fisher, MA, USA) and mirVana PARIS kits (Thermo Fisher), respectively. The cDNA was synthesized using PrimeScript RT reagent kits (TaKaRa, Shiga, Japan), and the concentration and purity of extracted RNA were determined using a UV spectrophotometer (FPMRC-SPECTRO-Nano, Fuguang Precision Instrument, Shanghai, China). RT-qPCR was performed using ChamQTM SYBR qRT-PCR MasterMix (Vazyme Biotech, Nanjing, China). The reaction conditions included an initial denaturation at 95 ^∘^C for 30 s, followed by 35 cycles of denaturation at 95 ^∘^C for 10 s, annealing at 60 ^∘^C for 30 s, and extension. The relative mRNA expression of the target genes was calculated using the 2^-^^ΔΔ^^Ct^ method with β-actin (*ACTB*) as an internal reference. The primer sequences used are provided in Table 1.

**Table 1:** RT-qPCR primer sequences

**Gene**	**Forward 5’-3’**	**Reverse 5’-3’**
*NLRP3*	AAGGCCGACACCTTGATATG	CCGAATGTTACAGCCAGGAT
*LC3B*	TTCAGGTTCACAAAACCCGC	TCTCACACAGCCCGTTTACC
*p62*	CCGTGAAGGCCTACCTTCTG	TCCTCGTCACTGGAAAAGGC
*BECN1*	GAAGTTTTCCGGCGGCTACC	CTCAGCCCCCGATGCTCTTC
*ACTB*	CATGTACGTTGCTATCCAGGC	CTCCTTAATGTCACGCACGAT

*NLRP3:* NOD-like receptor thermal protein domain associated protein 3; *LC3B*: Autophagy marker light chain 3B; *BECN1:* Beclin 1; *ACTB:* β-actin.

### Strategies for platelet transfusion therapy

Platelet transfusion therapy for all patients followed the technical specifications for clinical transfusion and the Guide to Clinical Decision Making for Blood Transfusion [24]. Platelet infusions were given to patients with the same ABO and RhD type. Each bag of platelets contained one therapeutic amount of platelets with a volume of 250–300 mL. Before infusion, 5 mg of dexamethasone was intramuscularly injected to prevent transfusion reactions. The infusion was completed within 30 min, starting with a slow infusion rate. If the patient did not experience any transfusion reaction, the infusion rate could be increased to the patient’s tolerance. Shaking during the infusion was done to prevent platelet aggregation and achieve the hemostatic peak [25].

### Evaluation of the efficacy of platelet transfusion

The corrected count increment (CCI) was used as the evaluation index of the treatment effect. CCI was calculated as follows:

CCI ═ (platelet difference before and after platelet infusion)× (total surface area/the number of transfused platelets).

 The evaluation criteria for CCI efficacy were as follows: if the 1 h CCI was greater than 7.5 × 10^9^/L or the 24 h CCI was greater than 4.5 × 10^9^/L, the platelet infusion was considered effective. Otherwise, it was considered ineffective [25].

### Isolation, culture, and grouping of the peripheral blood mononuclear cells (PBMCs)

Peripheral blood samples were obtained from healthy volunteers, and buffy coats were separated. Primary PBMCs were isolated using density gradient centrifugation with FIColL-Paque PLUS (GE Healthcare, Uppsala, Sweden), and platelets were removed. The isolated PBMCs without platelets were cultured in RPMI-1640 medium (Gibco, MA, USA) supplemented with 10% endotoxin-free bovine fetal serum (Lymphoprep, Paris, France), 100 U/mL penicillin, and 100 µg/mL streptomycin (Solarbio, Beijing, China) in a CO_2_ incubator at 37 ^∘^C. The culture medium was changed every two days, and cells at the logarithmic growth phase were collected for the experiment. For co-culture with platelets, PBMCs and platelets with different storage times were incubated in a ratio of 1:100 in 6-well plates for 48 h.

The PBMCs were divided into the following groups: the control group (not co-cultured with platelets), the d0 group (co-cultured with platelets preserved for 0–4 h), the d1 group (co-cultured with platelets preserved for 4 h–1 d), the d2-3 group (co-cultured with platelets preserved for 1 d–3 d), and the d4-5 group (co-cultured with platelets preserved for 3 d–5 d).

### Activation analysis of monocytes/macrophages, B cells, and T cells

After co-culturing PBMCs and platelets for 48 h, flow cytometry was performed to analyze the activation of monocyte/macrophage, B cells, and T cells. Monoclonal antibodies (MoAbs) were used to label T cells, B cells, and monocytes/macrophages with specific activation markers (Table 2). The expression of these markers was evaluated to assess the activation status [26].

**Table 2:** Monoclonal antibodies used for studying the cell surface markers

**Cell type**	**MoAb cell marker**	**Fluorochrome-conjugated**	**Manufacturers, clone number**
B cell	CD19	APC	Abcam, CB19 [58]
	CD69	FITC	BD Biosciences, FN50 [26]
	CD86	PE	Abcam, BU63 [59]
T cell	CD3	APC	BD Biosciences, UCHT1 [60]
	CD69	PE	BD Biosciences, FN50 [26]
	CD25	FITC	BD Biosciences, 2A3 [61]
Monocyte/macrophage	CD14	APC	BD Biosciences, MφP9 [26]
	CD86	PE	Abcam, BU63 [59]
	CD80	FITC	Abcam, RM80 [62]

CD19 is a B cell surface marker, CD69 and CD86 are B cell activation markers. CD3 is a T cell surface marker, CD69 and CD25 are T cell activation markers. CD14 is a monocyte/macrophage surface marker, CD86 and CD80 are monocyte/macrophage activation markers. MoAb: Monoclonal antibody; CD: Cluster of differentiation; APC: Allophycocyanin; FITC: Fluorescein isothiocyanate; PE: R-phycoerythrin.

### Ethical statement

This study was conducted in accordance with the principles of the Declaration of Helsinki. The ethics committee of The First Affiliated Hospital of Nanjing Medical University approved this study (approval no. 2022-SR-264), and informed consent was obtained from each participant.

### Statistical analysis

Statistical analyses and data plotting were performed using SPSS21.0 software (IBM Corp., Armonk, NY, USA) and GraphPad Prism 6.0 software (GraphPad Software Inc., CA, USA). The Shapiro–Wilk test was used to determine the normal distribution of the data. Normally distributed measurement data were presented as mean ± standard deviation (SD). The *t*-test was used for comparing data between two groups, while one-way analysis of variance (ANOVA) followed by Newman Keuls post hoc test was used for comparing data among multiple groups. Count data were presented as numbers and percentages, and the chi-square (χ^2^) test was used for comparing data between groups. Logistic regression was performed for multivariate analysis, and odds ratios (OR) with 95% confidence interval (CI) were calculated. A *P* value less than 0.05 was considered statistically significant.

## Results

### Comparative analysis of baseline data of apheresis platelets donors

The baseline data of AP donors in different storage time groups were compared. The analysis showed that there were no significant differences in age, sex, and times of blood donation among AP donors in each group (*P* > 0.05) (Table 3).

**Table 3:** Single factor analysis of blood donors affecting infusion effectiveness

	**Time (day)**				***χ^2^***	***P* value**
	**d0**	**d1**	**d2-3**	**d4-5**		
Age (years)					1.385	0.7091
<39	6	9	6	5		
≥39	7	6	8	3		
Sex					0.191	0.9790
Male	4	10	7	4		
Female	3	7	5	4		
Number of blood donations						
1	2	4	4	3	1.475	0.9612
2–9	6	5	4	4		
≥ 10	4	3	3	2		

### Changes of activation indices and thrombelastogram (TEG) parameters of apheresis platelets during preservation

The expression levels of the activation indicators CD62P and PAC-1 in APs were measured during the preservation period to investigate the effect of preservation time on the AP activity. Flow cytometry analysis revealed that the levels of CD62P and PAC-1 were reduced in the d1 group compared with the d0 group, while they were significantly enhanced in the d2-3 and d4-5 groups. Additionally, the levels of CD62P and PAC-1 in the d4-5 group were significantly higher than those in the d2-3 group (*P* < 0.01) (Table 4).

**Table 4:** Changes of platelet activation indices (CD62P and PAC-1) during AP preservation time

	**d0**	**d1**	**d2-3**	**d4-5**
CD62P (%)	19.24 ± 1.15	11.94 ± 3.83^**^	28.13 ± 6.95^##^	47.59 ± 10.74^ΔΔ^
PAC-1 (%)	0.97 ± 0.24	0.69 ± 0.20^**^	1.19 ± 0.39^##^	1.38 ± 0.46^ΔΔ^

Compared with d0, ^**^P < 0.01; compared with d1, ^##^*P* < 0.01; compared with d2-3, ^ΔΔ^*P* < 0.01. CD62P: P-selectin; PAC-1: Procaspase-activating compound 1; AP: Apheresis platelet.

TEG parameters were also examined during AP storage. The results showed that, as the preservation time increased, the R value (reflecting clotting time) of AP increased significantly, while the angle of clot formation (ANG) and the maximum clot firmness (MA) values decreased significantly (except for the MA value in the d1 group) (all *P* < 0.05). There was no significant difference in the coagulation time (*K*) value among the groups (all *P* > 0.05) (Table 5). These findings indicated that the activity of coagulation factors of AP, fibrinogen levels, and platelet aggregation in APs decreased as the preservation time was prolonged.

**Table 5:** Detection of TEG parameters during AP preservation time

	**d0**	**d1**	**d2-3**	**d4-5**
R (min)	5.91 ± 1.15	6.03 ± 1.41^**^	7.10 ± 1.69^**##^	8.99 ± 2.15^**##ΔΔ^
K (min)	1.08 ± 0.65	1.11 ± 0.25	1.16 ± 0.21	1.23 ± 0.53
ANG (∘)	80.24 ± 0.77	79.98 ± 0.54^**^	79.12 ± 0.55^**##^	77.24 ± 0.64^**##ΔΔ^
MA (mm)	84.38 ± 6.09	86.06 ± 4.07	83.52 ± 4.68^*^	79.61 ± 3.75^**##ΔΔ^

R respresents the reaction time, reflecting the comprehensive effect of coagulation factors involved in the initiation of coagulation. K is the time required from the end point of R to the tracing amplitude of 20 mm, it is the reaction rate of blood clot formation. ANG is the α angle, the angle between the tangent and the horizontal line from the formation point of the blood clot to the maximum curve radian of the tracing chart. MA represents maximum amplitude, reflecting the maximum intensity of blood clots. Compared with d0, ^*^P < 0.05, ^*^^*^P < 0.01; compared with d1, ^##^*P* < 0.01; compared with d2-3, ^Δ^^Δ^*P* < 0.01. TEG: Thrombelastogram; AP: Apheresis platelet.

### The inflammatory response of apheresis platelets increased with the preservation time

The levels of the inflammatory factors IL-1β, IL-6, TNF-α, and NOD-like receptor thermal protein domain associated protein 3 (*NLRP3*) expression in APs were measured during the preservation period using ELISA and RT-qPCR, respectively. The analysis revealed that there were no significant differences in the levels of IL-1β, IL-6, TNF-α, and *NLRP3* between the d1 and d0 groups (*P* > 0.05). However, compared with the d0 or d1 group, the levels of IL-1β, IL-6, TNF-α, and *NLRP3* in the d2-3 and d4-5 groups were elevated, and the levels in the d4-5 group were significantly higher than those in the d2-3 group (*P* < 0.01) (Table 6). These findings indicate that the inflammatory response of APs increased with the prolongation of preservation time.

**Table 6:** Detection of inflammatory factors (IL-1**β**, IL-6, TNF-**α**, and *NLRP3*) during AP preservation time

	**d0**	**d1**	**d2-3**	**d4-5**
IL-1β (pg/mL)	6.26 ± 0.51	5.88 ± 0.51	6.79 ± 0.82^**##^	8.18 ± 0.75^ΔΔ^
IL-6 (pg/mL)	3.98 ± 0.31	4.00 ± 0.25	5.15 ± 0.87^**##^	6.43 ± 0.46^ΔΔ^
TNF-α (pg/mL)	22.58 ± 7.88	22.19 ± 6.32	28.25 ± 9.15^**##^	30.62 ± 6.74^**##^
*NLRP3*	0.99 ± 0.27	1.04 ± 0.32	1.22 ± 0.36^**#^	1.53 ± 0.56^ΔΔ^

Compared with d0, ^*^^*^P < 0.01; compared with d1, ^#^*P* < 0.05, ^##^*P* < 0.01; compared with d2-3, ^ΔΔ^*P* < 0.01. IL-1β: Interleukin 1 beta; IL-6: Interleukin 6; TNF-α: Tumor necrosis factor--alpha; *NLRP3*: NOD-like receptor thermal protein domain associated protein 3; AP: Apheresis platelet.

### The autophagy level of apheresis platelets increased with the preservation time

The expression levels of autophagy-related genes (autophagy marker light chain 3B gene [*LC3B*], *p62*, and *Beclin 1* [*BECN1*]) in APs were measured during the preservation time using RT-qPCR. The results showed that compared to the d0 group, the levels of *LC3B* and *BECN1* in the d2-3 and d4-5 groups were reduced, while the levels of *p62* were increased. Additionally, the levels of *LC3B* in the d2-3 group and the levels of *LC3B* and *BECN1* in the d4-5 group were lower than those in the d1 group (*P* < 0.05), while the levels of *p62* were prominently higher than those in the d1 group. (Table 7). These findings suggest that the autophagy level of APs was enhanced with the preservation time.

**Table 7:** Detection results of autophagy-related genes (*LC3B*, *p62*, and *BECN1*) during AP preservation time

	**d0**	**d1**	**d2-3**	**d4-5**
*LC3B*	1.04 ± 0.29	1.00 ± 0.30	0.83 ± 0.09^**##^	0.76 ± 0.21^**##^
*p62*	1.00 ± 0.27	1.02 ± 0.34	1.15 ± 0.33^*^	1.24 ± 0.36^**##^
*BECN1*	1.00 ± 0.51	0.98 ± 0.27	0.81 ± 0.25^**^	0.79 ± 0.21^**##^

Compared with d0, ^*^P < 0.05, ^**^P < 0.01; compared with d1, ^##^*P* < 0.01. *LC3B*: Autophagy marker light chain 3B; *BECN1*: Beclin 1; AP: Apheresis platelet.

### The activation degree of immune cells stimulated by apheresis platelets increased with the preservation time

**Figure 1.**
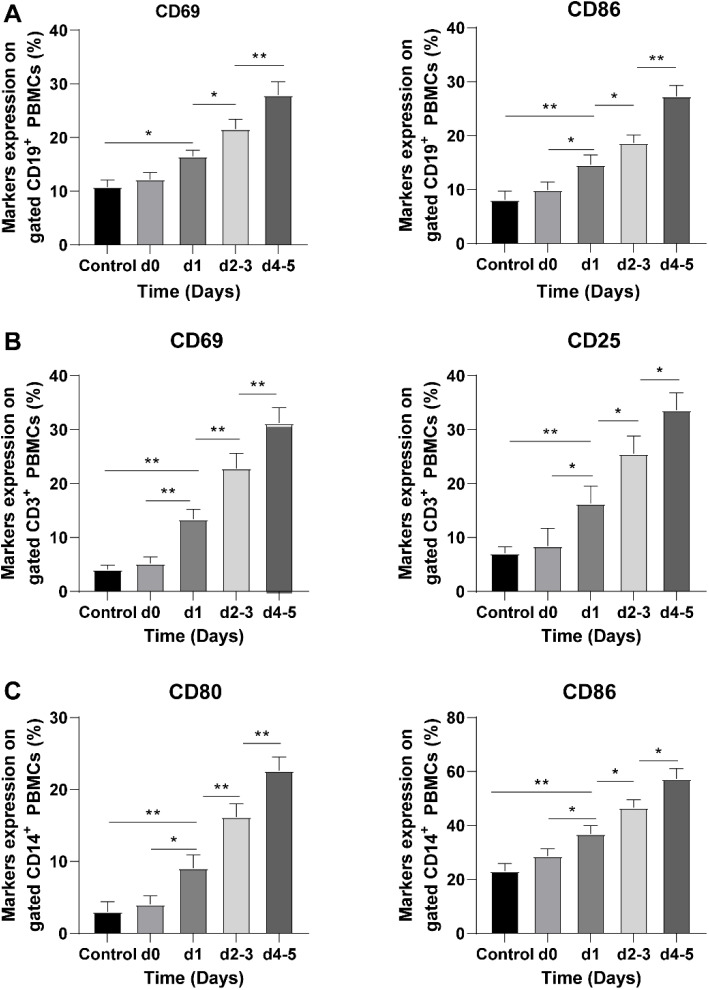
**Activation degree of immune cells stimulated by APs increases with preservation time.** APs at different preservation periods were co-cultured with PBMCs for 48 h (PLTs group), and PBMCs without platelets were cultured for 48 h as controls. (A) B cell activation markers CD69 and CD86 were analyzed using flow cytometry; (B) T cell activation markers CD69 and CD25 were analyzed using flow cytometry; (C) Monocyte/macrophage activation markers CD86 and CD80 were analyzed using flow cytometry. Measurement data are presented as mean ± SD (*n* ═ 3). Data comparison between two groups was performed using the *t*-test, and data comparison between multiple groups was performed using one-way ANOVA followed by a Newman Keuls post hoc test. *P < 0.05, **P < 0.01. APs: Apheresis platelets; PBMCs: Peripheral blood mononuclear cells; PLTs: Platelets; CD: Cluster of differentiation.

To evaluate the effect of APs stored for different durations on immune cell activation, PBMCs consisting of B cells, T cells, and monocytes/macrophages were co-cultured with APs for 48 h. Flow cytometry analysis was performed to assess the expression of specific activation markers on immune cells. The results showed that the expression of B cell activation markers CD69 and CD86 increased with the prolongation of AP preservation time (*P* < 0.05). Similarly, the expression of T cell activation markers CD69 and CD25 was enhanced with the increase of AP preservation time (*P* < 0.05). Additionally, the expression of monocyte/macrophage activation markers CD80 and CD86 was elevated with the prolongation of AP preservation time (*P* < 0.05) (Figure 1A–1C). Overall, these findings indicate that the activation degree of immune cells stimulated by APs increased with the preservation time.

### Analysis of clinical baseline characteristics and apheresis platelets-related indicators of ALL patients affecting the platelet transfusion effectiveness

The effectiveness of platelet transfusion was assessed in 280 patients with ALL, among which in 191 cases, the platelet transfusion was effective and in 89 cases, the platelet transfusion was ineffective, resulting in an effectiveness rate of 68.21%. The correlation between clinical baseline data, AP-related indicators, and platelet transfusion effectiveness in ALL patients was analyzed. The results demonstrated that the platelet antibody presence, the number of platelet transfusions, fever, splenomegaly, bleeding, and infection were significantly correlated with the effectiveness of platelet transfusion in ALL patients (all *P* < 0.05) (Table 8). Furthermore, platelet preservation time, IL-6, *p62*, and *BECN1*, among the related indices of APs, were also found to be correlated with the effectiveness of platelet transfusion in ALL patients (all *P* < 0.05) (Table 9).

**Table 8:** The analysis of clinical baseline characteristics in ALL patients affecting the efficacy of platelet transfusion

	**Effective (*n* ═ 191)**	**Ineffective (*n* ═ 89)**	**χ^2^**	***P* value**
Age (years)			2.593	0.107
<35	114 (59.69)	44 (49.44)		
≥35	77 (40.31)	45 (50.56)		
Sex			1.207	0.272
Male	95 (49.74)	38 (42.70)		
Female	96 (50.26)	51 (57.30)		
Blood type			0.158	0.984
A	47 (24.61)	20 (22.47)		
B	59 (30.89)	28 (31.46)		
O	41 (21.47)	20 (22.47)		
AB	44 (23.04)	21 (23.60)		
Platelet antibody			15.325	0.000
Positive	37 (19.37)	34 (38.20)		
Weakly positive	67 (35.08)	33 (37.08)		
Negative	87 (45.55)	22 (24.72)		
Number of platelet transfusions			16.350	0.001
1	39 (20.42)	5 (5.62)		
2∼5	93 (48.69)	41 (46.07)		
6∼10	50 (26.18)	31 (34.83)		
>10	9 (4.71)	12 (13.48)		
Fever			5.123	0.024
>37.2 ^∘^C	44 (23.04)	32 (35.96)		
≤37.2 ^∘^C	147 (76.96)	57 (64.04)		
Splenomegaly			5.476	0.019
Yes	24 (12.57)	21 (23.60)		
No	167 (87.43)	68 (76.40)		
Hemorrhage			5.097	0.024
Yes	28 (14.66)	23 (25.84)		
No	163 (85.34)	66 (74.16)		
Infection			4.364	0.037
Yes	42 (21.99)	30 (33.71)		
No	149 (78.01)	59 (66.29)		
Antibiotics			1.097	0.295
Yes	67 (35.08)	37 (41.57)		
No	124 (64.92)	52 (58.43)		

The values in the parentheses respresent percentages (%). ALL: Acute lymphoblastic leukemia.

**Table 9:** Analysis of AP-related indices affecting platelet transfusion efficacy

	**Effective (*n* ═ 191)**	**Ineffective (*n* ═ 89)**	***P* value**
Storage time			0.040
d0	53 (27.75)	13 (14.61)	
d1	55 (28.80)	23 (25.84)	
d2-3	47 (24.61)	27 (30.34)	
d4-5	36 (18.85)	26 (29.21)	
IL-1β	6.66 ± 1.06	6.84 ± 1.10	0.188
IL-6	4.62 ± 1.02	5.30 ± 1.17	0.000
TNF-α	25.80 ± 8.91	26.35 ± 7.63	0.615
*NLRP3*	1.54 ± 0.29	1.54 ± 0.25	0.906
*LC3B*	0.93 ± 0.29	0.86 ± 0.290	0.052
*p62*	1.07 ± 0.31	1.16 ± 0.40	0.039
*BECN1*	0.94 ± 0.28	0.81 ± 0.26	0.000

The numbers respresent mean (± SD) and n (%). AP: Apheresis platelet; IL-1β: Interleukin 1 beta; IL-6: Interleukin 6; TNF-α: Tumor necrosis factor-alpha; *NLRP3*: NOD-like receptor thermal protein domain associated protein 3; *LC3B*: Autophagy marker light chain 3B; *BECN1*: Beclin 1.

### Logistic multifactor regression analysis of platelet transfusion effectiveness in ALL patients

To further investigate the independent correlation between changes in inflammatory response and autophagy of AP during preservation and PTR in ALL patients, logistic multifactor regression analysis was performed. The effectiveness of AP transfusion in ALL patients was taken as the dependent variable, while platelet antibody presence, the number of platelet transfusions, fever, splenomegaly, bleeding, infection, platelet preservation time, IL-6, *LC3B*, *p62*, and *BECN1* (as indicated in Tables 8 and 9 with *P* < 0.05) were included as independent variables. After adjusting for platelet antibodies, the number of platelet transfusions, fever, splenomegaly, bleeding, and infection, the results showed that platelet preservation time, inflammatory index IL-6, and autophagy indices *p62* and *BECN*1 were independent risk factors affecting the effectiveness of platelet transfusion in ALL patients (all *P* < 0.05) (Table 10).

**Table 10:** Logistic multivariate regression analysis of the platelet transfusion effectiveness in ALL patients

	**β**	**SE**	**Wals**	*P*	**OR**	**95% CI**
Platelet antibody	−0.530	0.202	6.864	0.009	0.589	0.396∼0.875
Number of platelet transfusions	−0.251	0.053	22.336	0.000	0.778	0.701∼0.864
Fever	−1.145	0.287	15.933	0.000	0.318	0.181∼0.558
Splenomegaly	−0.929	0.419	4.912	0.027	0.395	0.174∼0.898
Hemorrhage	−0.743	0.406	3.350	0.067	0.476	0.215∼1.054
Infection	−1.015	0.354	8.211	0.004	0.363	0.181∼0.726
Storage time	0.749	0.296	6.406	0.011	2.115	1.184∼3.776
IL-6	−1.015	0.282	12.964	0.000	0.362	0.208∼0.630
*p62*	−0.882	0.501	3.095	0.079	0.414	0.155∼1.106
*BECN1*	1.719	0.672	6.539	0.011	5.580	1.494∼20.839

ALL: Acute lymphoblastic leukemia; IL-6: Interleukin 6; *BECN1*: Beclin 1.

## Discussion

ALL is a malignancy characterized by the presence of malignant cells originating from B and T lymphoid sources [27]. Thrombocytopenia, a common clinical symptom in various hematologic malignancies, particularly in patients with ALL, necessitates platelet transfusion to prevent severe bleeding episodes [5, 8, 28]. However, PTR is a phenomenon observed in many patients following platelet infusion [20]. Our study findings revealed that the inflammatory response and autophagy levels of APs, as well as the activation degree of immune cells stimulated by APs, increased with the duration of AP preservation. Moreover, we identified the AP preservation time, IL-6, *p62*, and *BECN1* as independent risk factors for PTR in patients with ALL.

A total of 280 patients with ALL were enrolled in the study, and the APs used for platelet transfusion were obtained from 44 blood donors. Platelet transfusion is commonly employed as a therapeutic measure for managing thrombocytopenia-related bleeding complications [6]. However, in many regions, including China, APs have a short storage time of up to five days due to the increased risk of bacterial contamination [15, 29]. The efficacy of clinical platelet transfusion is influenced by platelet activation and survival [30]. Consequently, to investigate the impact of preservation time on AP activity, we initially assessed the expression of activation indicators (CD62P and PAC-1) in APs during the preservation period. Our study observed increased levels of AP activation indicators (CD62P and PAC-1) over time. These findings align with our conclusions, as platelets within APs units undergo damage during storage, leading to accelerated platelet activation and release responses [31]. This, in turn, affects platelet aggregation and impairs the hemostatic function of platelets [32]. To comprehensively evaluate the hemostasis of whole blood, we measured various thromboelastography parameters of APs during the preservation period [33]. As anticipated, our results demonstrated a decrease in coagulation factor activity, fibrinogen levels, and platelet aggregation with increasing preservation time.

Prolonged storage time of platelets has been associated with various adverse transfusion reactions, particularly an increased inflammatory response [34]. Intriguingly, our study revealed elevated levels of inflammatory cytokines (IL-1β, IL-6, and TNF-α) and *NLRP3* with the duration of AP preservation. Previous research has reported the production of inflammatory cytokines, such as TNF-α, IL-1β, and IL-6, during platelet storage, which is linked to transfusion-related adverse reactions [35]. Furthermore, long-term storage of APs has been associated with a higher incidence of inflammatory transfusion-related adverse events [36]. In summary, the inflammatory levels of APs were found to increase with the duration of preservation.

Autophagy, the primary mechanism of intracellular degradation and recycling in eukaryotes, plays a crucial role in maintaining cellular homeostasis by breaking down aged, damaged, and degraded organelles and proteins [37]. Autophagy is closely intertwined with various physiological and pathological processes, including host defense, cell survival, aging, autoimmune diseases, and neurodegenerative diseases [38–40]. Although the understanding of autophagy in anucleate cells is still incomplete, autophagy-related gene transcripts and proteins have been detected in platelets [41, 42]. Autophagy has been linked to erythropoiesis and hematologic diseases, and electron microscopy studies have revealed structures resembling autophagosomes in platelets from benign tumors, suggesting a close association between autophagy and platelets [43–45]. Accumulating evidence suggests that autophagy mechanisms exist in platelets and can influence platelet activation, adhesion, release, and aggregation, and the processes of platelet hemostasis and thrombosis, which are critical for maintaining platelet functions [46–48]. In our study, the levels of autophagy-related proteins (LC3B, p62, and BECN1) were found to increase during the preservation period of AP. BECN1, p62, and LC3B are considered fundamental markers of autophagy, and their expression is up-regulated during autophagy [49]. Autophagy occurred during the storage of APs [30]. Overall, our findings suggest that the autophagy level of APs is enhanced as the storage time increases.

Platelets play a critical role in immune responses, and blood transfusion can potentially contribute to the progression of ALL through its immunomodulatory effects [50, 51]. PBMCs, comprising B cells, T cells, and monocytes/macrophages, are utilized for the treatment of immunodeficiency diseases, metabolic genetic diseases, and malignant hematological diseases, by enriching peripheral blood stem cells, and they are also crucial for evaluating immune responses [52]. Therefore, in our study, we co-cultured APs with PBMCs to investigate the impact of APs on the activation of immune cells during different storage periods. Interestingly, we observed an increase in the expression of B cell activation markers (CD69/CD86), T cell activation markers (CD69/CD25), and monocyte/macrophage activation markers (CD80/CD86) after the co-culture at days 2–3 and days 4–5. In summary, the activation degree of immune cells stimulated by APs was enhanced with the duration of storage.

PTR represents a significant clinical complication associated with platelet infusion [20, 53]. In recent years, the incidence of PTR has been on the rise, and in severe cases, it can even lead to death [54]. In our study, we found a transfusion effectiveness rate of 68.21% in ALL patients receiving APs, indicating the need for improvement in the efficiency of platelet transfusion. Previous studies have identified factors such as blood incompatibility, long storage time, and treatment (e.g., plasma removal and pathogen inactivation) as causes of ineffective platelet transfusion [55]. Additionally, infection, fever, increased transfusion frequency leading to platelet antibodies synthesis, as well as splenomegaly and bleeding resulting in platelet consumption and loss, can all impact the effectiveness of platelet transfusion [56, 57]. Moreover, platelet autophagy activity has been found to be inversely correlated with clinical platelet transfusion efficacy [30]. Consistent with the aforementioned studies, our investigation revealed significant correlations between platelet antibody presence, number of platelet transfusions, fever, splenomegaly, bleeding, and infection (as clinical indicators) and platelet preservation time, IL-6, *p62*, and *BECN1* (as AP-related indicators) with the effectiveness of platelet transfusion in ALL patients. Prior studies have already identified fever and infection as independent risk factors for PTR in leukemia patients [54]. Through logistic multivariate regression analysis, our study made a novel finding by demonstrating that after adjusting for platelet antibodies, number of platelet transfusions, fever, splenomegaly, bleeding, and infection, the preservation time of APs, IL-6, p62, and *BECN1* emerged as independent risk factors influencing PTR in ALL patients.

## Conclusion

In summary, our study demonstrated that inflammatory responses, autophagy levels, and the activation of immune cells by APs increased with the duration of preservation. Moreover, AP preservation time, IL-6, p62, and *BECN1* were identified as independent risk factors for PTR in ALL patients. It is worth noting that while qRT-PCR is a sensitive technique commonly used for gene expression analysis, it detects mRNA levels and may not always correlate with protein expression. Factors, such as miRNA binding, epigenetic modifications, and protein processing, can lead to inconsistencies between mRNA and protein levels. Ideally, western blot assays should be conducted to validate the qRT-PCR results. However, due to time and financial constraints, we did not perform western blot assays in this study, which represents a limitation. Future research should aim to validate and explore the expression of autophagy-related genes using the western blot analysis. Additionally, our study had a relatively small sample size of ALL patients and a relatively short observation time period. Further research is needed to investigate the molecular mechanism underlying the effects of changes in inflammatory factors and autophagy on PTR during AP preservation. Multi-center studies with larger sample sizes are warranted to enhance the credibility of the results. Moreover, a deeper understanding of the molecular mechanisms influencing PTR during AP preservation is necessary.
